# Mechanisms underlying the protective effects of San‐Bai decoction against UV radiation on the skin

**DOI:** 10.1111/ics.70093

**Published:** 2026-03-23

**Authors:** Rui Zhang, Hong Zhang, Chengming Zhang, Wencai Jiang, Luonan Chen, Yimei Tan, Ying Zou, Xuelan Gu

**Affiliations:** ^1^ Department of Skin & Cosmetic Research, Shanghai Skin Disease Hospital, School of Medicine Tongji University Shanghai China; ^2^ NMPA Key Laboratory for Monitoring and Evaluation of Cosmetics Shanghai China; ^3^ Professional Technical Service Platform for Clinical Evaluation of Skin Health Related Products Shanghai Science and Technology Commission Shanghai China; ^4^ Unilever R&D Shanghai China; ^5^ International Research Center for Neurointelligence, The University of Tokyo Institutes for Advanced Study The University of Tokyo Tokyo Japan; ^6^ School of Mathematical Sciences and School of AI Shanghai Jiao Tong University Shanghai China; ^7^ Key Laboratory of Systems Biology, Shanghai Institute of Biochemistry and Cell Biology, Center for Excellence in Molecular Cell Science Chinese Academy of Sciences Shanghai China

**Keywords:** differential network, network pharmacology, reconstructed living skin equivalent model, Sai‐Bai decoction, skin lightening

## Abstract

**Background:**

San‐Bai decoction (SBD), as a classic TCM formula, has been administered orally to support skin health with a long record history dating back to the Ming Dynasty in China. However, the protective efficacy of SBD through topical application on skin remains unclear.

**Purpose:**

We aimed to evaluate the effectiveness of SBD in protecting skin against UV radiation through an integrated in silico–in vitro–in vivo framework and elucidate its underlying mechanisms using a systems biology approach.

**Methods:**

A public database was used to construct a network diagram linking SBD's active chemical components with corresponding potential target genes for network pharmacology analysis. Skin lightening efficacy was evaluated in vitro using a pigmented 3D skin model, while in vivo protective effects were assessed in a double‐blind, placebo‐controlled clinical study. Transcriptomic analysis using the microarray platform was conducted to explore underlying mechanisms among control, UV exposure and SBD treatment groups.

**Results:**

Network pharmacology analysis implicated over 800 effective targets regulated by SBD, with Gene Ontology enrichment analysis suggesting that they were involved in oxidative response and immune regulation. USBT2627, the aqueous extract of SBD, displayed skin lightening effect in a pigmented living skin equivalent model. Compared with the placebo, pretreatment with 5% USBT2627 effectively protected skin from UV damage, increasing L* values (*p* = 0.048) and ITA° (*p* = 0.022), and reducing erythema (*p* = 0.016). USBT2627 pretreatment also showed a mild effect on a* reduction (*p* = 0.052). Transcriptomic analysis indicated that USBT2627 regulated multiple pathways related to skin pigmentation, such as ET‐1 signalling and pigmentation signalling pathways, as well as oxidation response and cytokine signalling pathways.

**Conclusions:**

USBT2627 suppresses melanin accumulation in vitro and protects skin against UV exposure in both in vitro and in vivo models; multiple components, targets and pathways of SBD act together to confer its protective effect against UV exposure. These findings highlight a significant potential of topical USBT2627 in protecting skin against UV exposure. Additionally, this study showcases the value of a systems network approach in elucidating the mechanisms of this herbal blend, offering valuable references for further research aimed at developing TCM‐based products.

## Graphical abstract

Combining conventional differential expression analysis with differential network analysis offers a promising strategy to uncover the mechanistic basis of the holistic benefits provided by herbal blends. Our findings demonstrated that San‐Bai decoction modulates multiple pathways—not only those related to skin pigmentation, such as ET‐1 signalling, but also those involved in oxidative response and cytokine signalling.

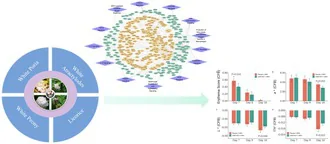

## Résumé

**Contexte:**

La décoction San‐Bai (DSB), formule classique de la médecine traditionnelle chinoise (MTC), est administrée par voie orale pour soutenir la santé de la peau et bénéficie d’un long historique d’utilisation remontant à la dynastie Ming en Chine. Toutefois, son efficacité protectrice, lorsqu’elle est appliquée localement sur la peau, reste à démontrer.

**Objectif:**

Nous avons cherché à évaluer l’efficacité de la DSB dans la protection de la peau contre les rayonnements UV au moyen d’un cadre intégré *in silico–in vitro–in vivo* et à élucider ses mécanismes d’action sous‐jacents à l’aide d’une approche de biologie des systèmes.

**Méthodes:**

Une base de données publique a été utilisée pour créer un diagramme de réseau reliant les composants chimiques actifs de la DSB aux gènes cibles potentiels correspondants, dans le cadre d’une analyse de pharmacologie des réseaux. L’efficacité éclaircissante de la peau a été évaluée in vitro à l’aide d’un modèle de peau 3D pigmentée, tandis que les effets protecteurs in vivo ont été étudiés dans le cadre d’un essai clinique en double aveugle contrôlé contre placebo. Une analyse transcriptomique au moyen d’une plateforme de puces à ADN (microarray) a été réalisée afin d’explorer les mécanismes sous‐jacents au sein des différents groupes : contrôle, exposé aux UV et traité par DSB.

**Résultats:**

L’analyse de pharmacologie des réseaux a mis en évidence plus de 800 cibles fonctionnelles régulées par la DSB, l’analyse d’enrichissement en ontologie des gènes suggérant leur rôle dans la réponse au stress oxydatif et la régulation immunitaire. L’USBT2627, extrait aqueux de la DSB, a montré un effet éclaircissant dans un modèle de peau vivante équivalente pigmentée. Comparativement au placebo, un prétraitement par USBT2627 à 5 % a protégé efficacement la peau contre les dommages induits par les UV, avec une augmentation des valeurs L* (p = 0,048) et de l’ITA° (p = 0,022), ainsi qu’une diminution de l’érythème (p = 0,016). Le prétraitement par USBT2627 a également montré une légère réduction du paramètre a* (p = 0,052). L’analyse transcriptomique a indiqué que l’USBT2627 régulait plusieurs voies liées à la pigmentation cutanée, notamment la voie de signalisation de l’ET‐1 et les voies de signalisation de la pigmentation, ainsi que des voies liées à la réponse au stress oxydatif et à la signalisation des cytokines.

**Conclusions:**

L’USBT2627 inhibe l’accumulation de mélanine in vitro et protège la peau contre l’exposition aux UV dans des modèles in vitro et in vivo ; les multiples composants, cibles et voies de la DSB agissent de manière synergique pour lui conférer son effet protecteur contre les UV. Ces résultats mettent en évidence le fort potentiel de l’USBT2627 en application topique pour la protection cutanée contre les rayonnements UV. Cette étude démontre en outre l’intérêt d’une approche de réseau des systèmes pour élucider les mécanismes de ce mélange à base de plantes, et fournit des éléments de référence utiles pour de futures recherches visant au développement de produits fondés sur la MTC.

## INTRODUCTION

Traditional Chinese medicine (TCM) has been practiced for treating skin problems through oral intake or topical application for thousands of years. San‐Bai decoction (SBD), a classic TCM formula for skin brightening, was first recorded in ‘Yi Xue Ru Men’ by Chan Li 600 years ago [1]. Recent studies have indicated that SBD may help to improve skin tone through reducing pigmentation and inhibiting tyrosinase [2, 3]. The flavonoid components present in SBD were identified as responsible for its biological activity [4]. Yet, most research has been confined to in vitro experiments, lacking clinical evidence and thorough investigation of its skin‐lightening mechanisms [5].

The complexity inherent in traditional medicine, characterized by its ‘multi‐ingredient, multi‐compound, multi‐target, multi‐function’ pattern, requires a holistic analysis approach. Network pharmacology, an emerging research paradigm shifting from single compound‐single target to multi‐compound‐multi targets, was proposed to decipher the underlying mechanism [6]. This approach was widely used in drug discovery [7] to decipher the combination principles of herbal formulae. Meanwhile, conducting transcriptomic studies using samples obtained from human clinical trials, including analyses of differential gene expression and gene–gene interaction networks, is essential for gaining a comprehensive understanding of the efficacy of traditional medicine.

Skin pigmentation is mainly due to melanin production and deposition, which is affected by various factors, such as ultraviolet (UV) radiation. UV irradiation can induce a series of biological processes like DNA damage, generation of reactive oxygen species, oxidative stress, inflammation and may further lead to skin ageing, pigmentation, etc. Depigmentation agents such as vitamin C, resveratrol have antioxidant effects. More ingredients and traditional medicines are used for antioxidant and melanin inhibition, such as licorice, green tea, mulberry [8], although comprehensive mechanisms of depigmentation of traditional medicines are rarely studied. In this study, network pharmacology was employed to unravel the interactions of SBD from chemical and biological perspective and discover the potential function on skin. The UVB‐irradiated pigmented living skin equivalent model was leveraged to evaluate the efficacy of aqueous extract of SBD (USBT2627) in UVB‐induced pigmentation. Furthermore, the clinical study was performed to demonstrate USBT2627 efficacy in alleviating skin phenotypic change after UV irradiation, particular skin lightening related parameters. Additionally, a differential network analysis was conducted to unravel the mechanistic basis of USBT2627's skin protection efficacy from a holistic point of view.

## MATERIALS AND METHODS

### San‐Bai decoction (USBT2627) preparation

Dried raw materials of atractylodes, poria cocos, white peony and licorice were purchased from Shanghai Huayuan Caotang Company (Shanghai, China).

Raw material (mix of four ingredients) was crushed into small pieces and soaked in 10‐fold volume (w/v) of deionized water for 30 min, then were reflux extracted for 30 min. After filtered through 16‐layer gauzes, the residues were reflux extracted for 30 min in another 10‐fold volume (w/v) of deionized water again. The extract solution was freeze‐dried to obtain aqueous extract powder.

### 3D skin model treatment and melanin content measurement

UV‐challenged pigmented living skin equivalent models (pLSE, MelaKutis®, purchased from Guangdong Biocell Biotechnology, Guangdong, China) were used to evaluate the skin lightening efficacy of USBT2627 [9]. The pLSE model was irradiated with 50 mJ/cm^2^ UVB once daily [10]. Two experimental studies were conducted separately (Figure S1). In the first study, pLSE models were treated with three different doses of the USBT2627 through systemic application route for three days, the concentrations of the USBT2627 were 100 ppm, 200 ppm and 300 ppm, respectively. Skin models were collected 24 h post the last treatment for further analysis. In the follow‐up study, USBT2627 (300 ppm) was applied on the surfaces of the pLSE models daily after UVB irradiation for three days, starting from the fourth day. And then skin models were collected on day 6 for further analysis. Each group had three replicates. The melanin content measurement followed the protocol in the previous study [11].

### Clinical study

#### Clinical study design

In this single‐center, randomized, double‐blind, placebo‐controlled clinical trial, 23 healthy Chinese participants were recruited. Eligible participants were between 35 and 45 years old, had Fitzpatrick skin type III [12]. Subjects were recruited at Shanghai Skin Disease Hospital, Shanghai, China. Ethical approval was obtained from the Ethics Committee (Ethics No. 012 (2017)). All reagents utilized in this study are listed in the ‘Catalogue of Cosmetic Ingredients’ (2015 edition), as documented by the China Food and Drug Administration. The use of these reagents adheres strictly to the stipulated concentration limits. Informed consent was obtained from all participating volunteers, who were fully informed about the study's procedures. 5% of USBT2627 was formulated into a skin moisturizer as the test formula (USBT2627‐containing formula); the placebo formula was prepared as well.

This study included two parts: (1) phenotype study: assessing the efficacy of USBT2627 on UV‐induced skin model and (2) mechanism study: exploring the mechanistic insights into UV protection efficacy of USBT2627 (Figure 1). The left and right buttocks of each participant were randomly designated; a total of seven application areas were allocated (Figure S2). The study lasted 16 days, with minimal erythema dose (MED) identified on day 1 (Supplementary Methods).

**FIGURE 1:**
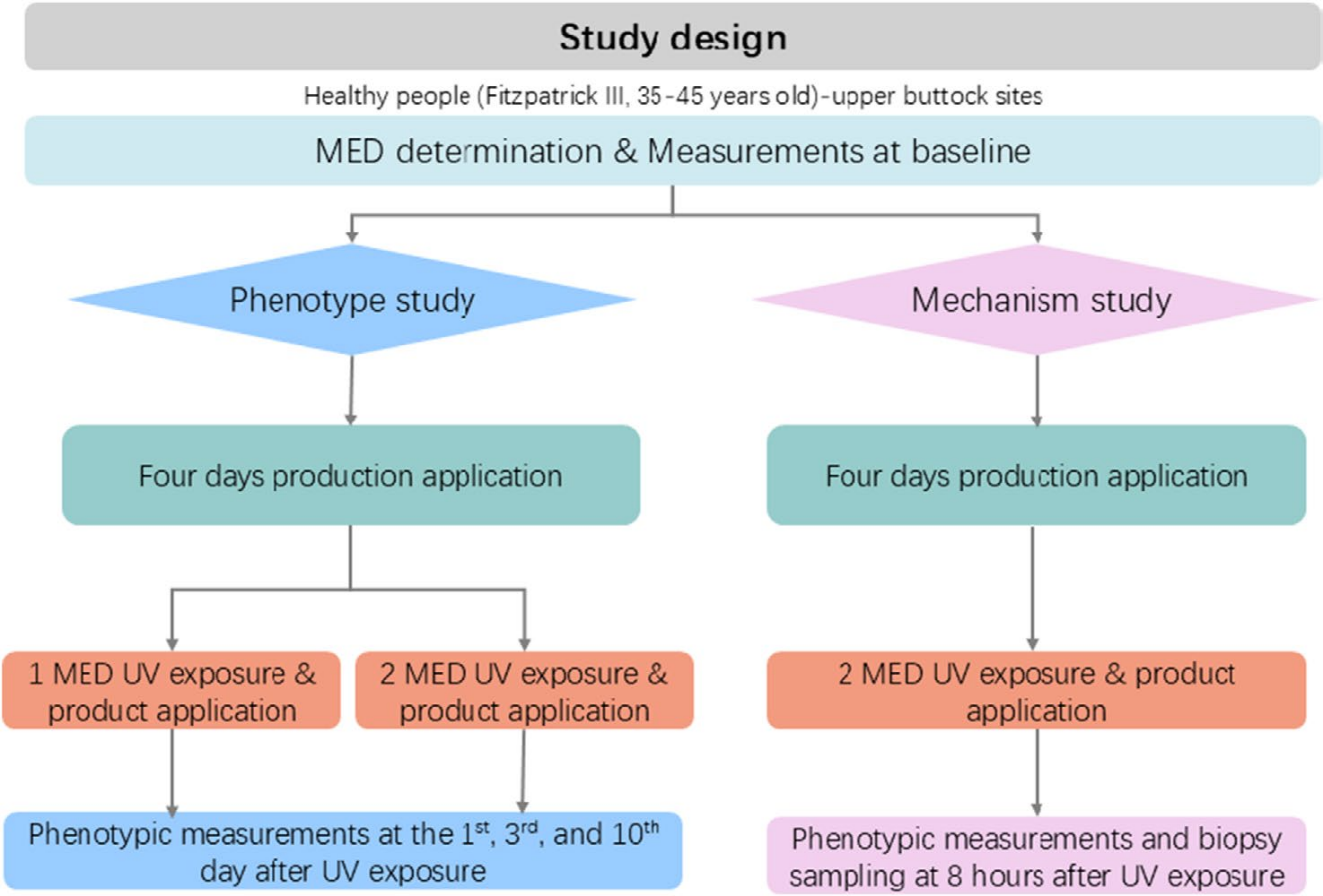
Scheme illustration of the clinical study design. The phenotype study was designed for efficacy assessment. The mechanism study was designed for underlying mode of action exploration through differential network analysis.

In the phenotype study, four areas (1.5 cm × 1.5 cm) were delineated, and two UV irradiation dosages (1 MED and 2 MED of combined UVA and UVB) (Supplementary Methods) were explored. According to a pre‐established randomization plan, each subject received the USBT2627‐containing formula and the placebo on the application areas, formulas were applied daily from day 2 to day 5 at a dose of 2 mg/cm^2^. On day 6, investigational areas were exposed to UV (1 MED or 2 MED) and applied by the last application of the product 10 min post UV irradiation. Visual and instrumental assessments were performed on days 7, 9 and 16, corresponding to the 1st, 3rd and 10th day post UV irradiation (Figures 1 and S3, Supplementary Methods).

In the mechanism study, three sites were designated for the USBT2627‐containing formula group, placebo group and UV exposure group. The USBT2627‐containing formula or placebo was randomly applied to the allocated areas daily from day 2 to day 5. On day 6, application areas were exposed to 2 MED UV irradiation, followed by the last treatment, either the test prototype or the placebo. The remaining area was exposed to 2 MED UV only and served as a reference for comparison. Biopsy samples were collected via 2 mm punch (2 mm, 33–31. Disposable Biopsy Punch. Integra® Miltex®, Avantor, PA, USA) at 8 h post UV exposure. Visual and instrumental assessments were performed before punching the biopsy (Figure 1, Figure S3). Biopsies were rinsed with buffer to remove possible blood, removed fat tissue and stored in RNAase‐free buffer until further analysis. Follow‐up observations were conducted to monitor skin colour and wound healing.

#### Clinical measurement

Skin colour at each of the assigned sites was measured at a single location using a Minolta Spectrophotometer (CM‐2600d, Minolta Camera Co., Ltd., Osaka, Japan). The colour values were expressed in the CIELAB colour space, utilizing the parameters L*, a*, b*, individual typology angle (ITA°) was calculated by

$${\mathrm{ITA}}^{{}^{\circ}}=\arctan\frac{\left(L^{*}-50\right)}{b^{*}}\times \frac{180}{\pi }$$

Concurrently, at each clinical visit, skin erythema at each of the assigned sites was evaluated by trained clinical specialists on a 4‐point ordinal scale (Table S1).

### Network pharmacology analysis

Compounds and targets information of each ingredient of USBT2627 were retrieved from the Traditional Chinese Medicine Systems Pharmacology (TCMSP) database and analysis platform [13]. The compound and target interaction network were visualized using Cytoscape (version 3.8.1).

### Transcriptomics data analysis

Transcriptomics profiling of biopsy samples was performed using microarray platform (Shanghai Biotechnology Corporation, Shanghai, China). RNA was extracted using RNeasy micro kit (Cat#74004, QIAGEN, GmBH, Germany). RNA quality was measured using Agilent Bioanalyzer 2100 (Agilent Technologies, Santa Clara, CA, US). Total RNA was amplified and labelled by Low Input Quick Amp Labeling Kit, One‐Colour (Cat. # 5190‐2305, Agilent Technologies, Santa Clara, CA, US). Labelled cRNAs was purified by RNeasy mini kit (Cat. # 74 106, QIAGEN, GmBH, Germany). Samples were then conducted microarray profiling using Agilent SurePrint G3 Human Gene Expression Microarray (Agilent Technologies, Santa Clara, CA, US).

Differential network analysis was performed using the method developed by Yu et al. [14]. Genes with differential gene expression, differential expression variance, and gene pairs with differential expression covariance were identified first. A differential score based on the above three indicators was integrated to construct the differential network (Figure S4). Function analysis of identified genes was conducted using Ingenuity Pathway Analysis (QIAGEN, GmBH, Germany) and R package clusterProfiler (version 4.12.6).

### Statistical analysis

In this study, quantitative data were first assessed for quantitative data, and data meeting assumptions were analysed using the paired Student's *t*‐test. For quantitative data not adhering to parametric assumptions, as well as for qualitative data, the Wilcoxon matched‐pairs signed‐rank test was employed. The threshold for statistical significance was set at *p* < 0.05. All relevant statistical analyses were performed using R version 4.4.1.

## RESULTS

### Holistic function of San‐Bai decoction through network pharmacology approach

Public domain information for individual ingredients of San‐Bai decoction was extracted including the compounds naturally present in individual herbal ingredients and potential regulated gene/proteins of compounds (Figure 2). Generally, 454 compounds were reported to be contained in San‐Bai decoction. Limited compounds were shared by any two ingredients of San‐Bai decoction. 286 of them were reported to regulate more than 800 genes/proteins and most of the targets were commonly regulated by compounds from different ingredients (Table 1). Gene ontology enrichment analysis showed that these targeted features were involved in oxidative response, immune regulation, etc. The public domain information presented the holistic effects of the herbal mix but with limitation for skin‐specific functions.

**FIGURE 2:**
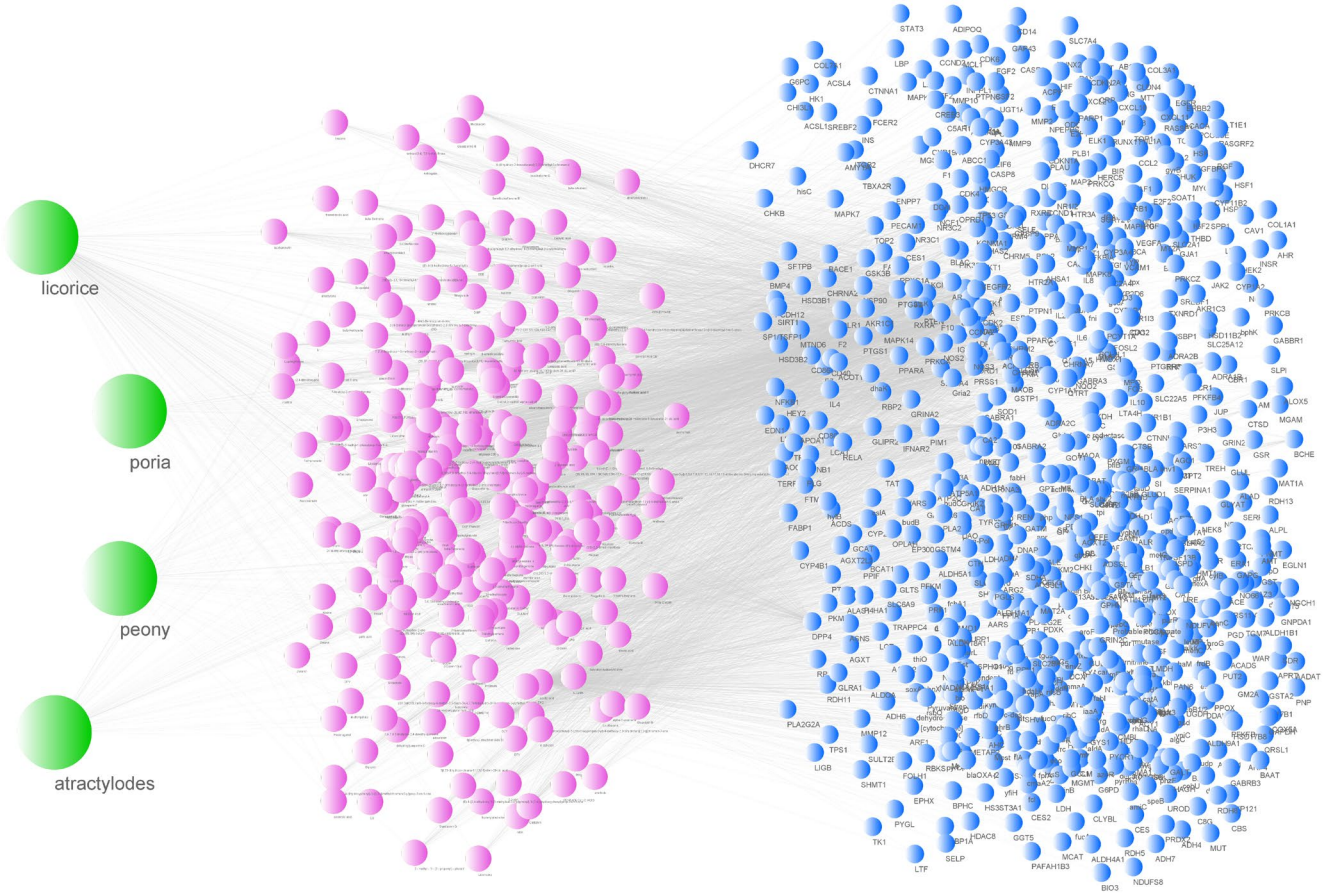
Visualization of the herb‐compound–target interaction retrieved from TCMSP database. Green: natural ingredients (poria, licorice, peony and atractylodes). Purple: compounds (Edge: compound contained in the linked ingredients). Blue: potential targets of compounds.

**TABLE 1:** The number of compounds and targeted features for San‐Bai decoction.

Herb name	Number of compound	Number of links	Number of targets
Peony	85	1225	653
Poria	34	228	85
Atractylodes	55	1006	270
Licorice	280	3169	315
Total	454 (10)^a^	5628	845

^a^ Number within the bracket is the number of shared compounds.

### USBT2627 reduced melanin content in the pLSE model

UVB irradiation significantly induced an increase in melanin content. The systemic application of USBT2627 significantly reduced the melanin content and showed a dose–response pattern (Figure 3a). Results of melanin reduction in the follow‐up study showed that the topical application of 300 ppm USBT2627 significantly decreased melanin production by 25.7%, as compared with the UVB group and performed a comparable potency with kojic acid (Figure 3b).

**FIGURE 3:**
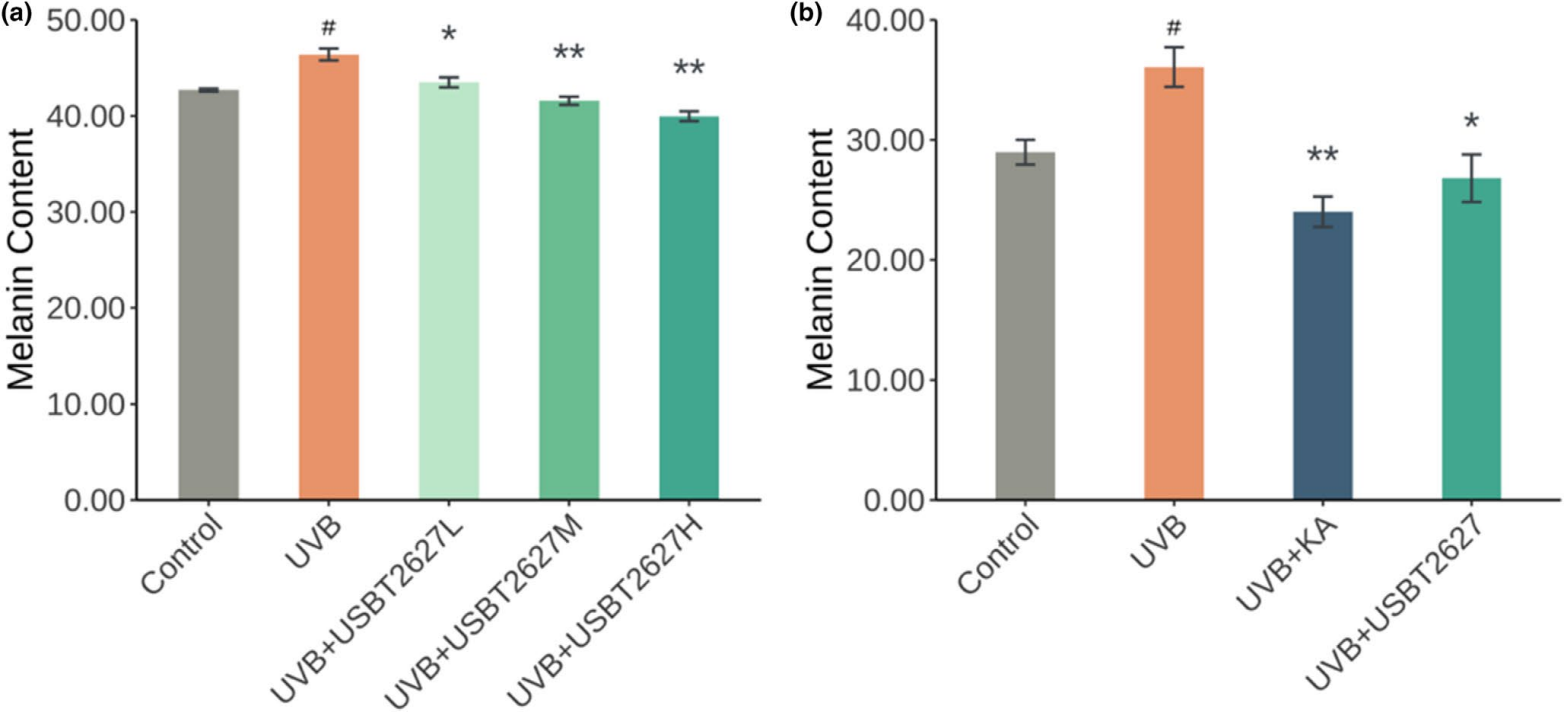
Melanin inhibition efficacy of USBT2627 on UVB challenge pLSE model. (a) The efficacy in melanin inhibition of systemic application of USBT2627 with different doses. (b) The efficacy in melanin inhibition of topical application of high‐dose USBT2627 and kojic acid. ^#^ *p* < 0.05 as compared with control group. *p < 0.05 as compared with UVB group. **p < 0.001 as compared with UVB group. KA, kojic acid. USBT2627‐L: low dose (100 ppm) of USBT2627, USBT2627‐M: medium dose (200 ppm) of USBT2627, USBT2627‐H: high dose (300 ppm) of USBT2627. All values are mean ± SE (*n* = 3). SE, standard error.

### USBT2627 effectively alleviated L*, ITA° changes and redness after UVB exposure in vivo

The UV absorption profile indicated that USBT2627 did not directly block UV (Figure S5). Meanwhile, the irritation patch test showed that there was no irritation of two formulas (Supplementary). In the clinical study, both 1 MED and 2 MED UV irradiation induced significant changes in erythema score, a*, L*, and ITA° values over time under either USBT2627‐containing formula or placebo application (Figure 4). Compared with the baseline, the erythema score and the a* value reached the peak on day 7 (the first day after irradiation), and then gradually decreased on day 9 and day 16, and 2 MED UV irradiation induced a stronger change than 1 MED UV irradiation (Figure 4a,b). While the L* and ITA° values decreased on day 7, and then gradually increased on day 9 and day 16 under 1 MED irradiation. However, L* and ITA° values consistently decreased under 2 MED UV irradiation and decreased compared with 1 MED UV irradiation (Figure 4c,d).

**FIGURE 4:**
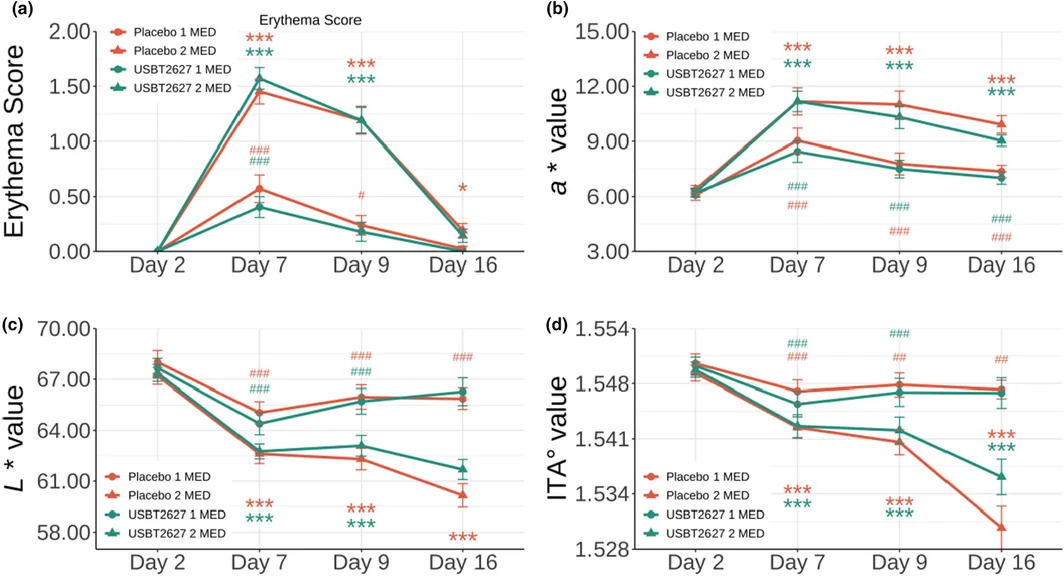
Skin appearance changes before and after UV irradiation. (a) Changes of erythema scores at different timepoints. (b) Changes of a* values at different timepoints. (c) Changes of L* values at different timepoints. (d) Changes of ITA° values at different timepoints. *p < 0.05, ***p < 0.001 as compared with baseline (day 2) under 2 MED UV irradiation. ^#^ *p* < 0.05, ^##^ *p* < 0.01, ^###^ *p* < 0.001 as compared with baseline (day 2) under 1 MED UV irradiation. All values are mean ± SE. SE, standard error.

Furthermore, the changes from the baseline (ΔL*, ΔITA°, and Δa*) were compared between the USBT2627‐containing formula and the placebo group. Under 1 MED UV irradiation, the USBT2627‐containing formula group had a smaller erythema score on the first day post UV exposure (*p* = 0.016) (Figure 5a). Under 2 MED UV irradiation, there was a significant difference between the USBT2627‐containing formula and placebo group, where the USBT2627‐containing formula group showed a smaller change of L* (*p* = 0.048) on day 16 compared with baseline, similar result for ITA° (*p* = 0.022) (Figure 5b,c). The change of a* showed a similar tendency but with a mild significance (*p* = 0.052) (Figure 5d). Under 1 MED irradiation, no significant differences in ΔL*, ΔITA° and Δa* were observed between groups (Figure S5).

**FIGURE 5:**
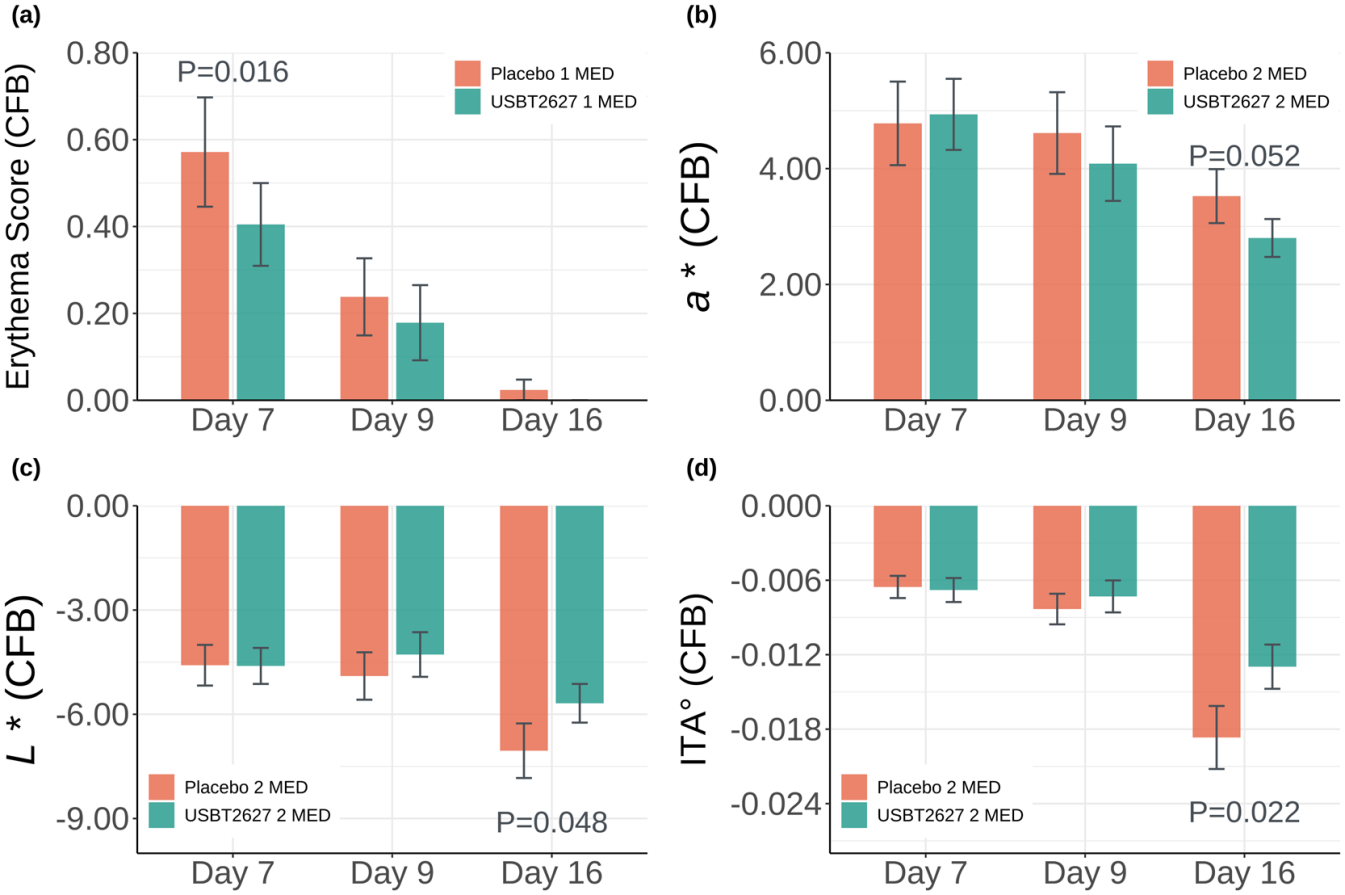
Skin protection efficacy of USBT2627 in the clinical study. (a) Change from baseline (CFB) in erythema scores over time under l MED irradiation. (b) CFB in a* values over time under 2 MED irradiation. (c) CPB in L* values over time under 2 MED irradiation. (d) CPB in ITA° values over time under 2 MED irradiation. Compared with the placebo group, *p* values that are statistically significant or approaching significance have been clearly indicated in the figures. All values are mean ± SE. SE, standard error.

### The holistic benefit of USBT2627 in skin formulation through differential network analysis of transcriptomic data

To further understand the mechanism of the skin lightening effect of USBT2627 from a holistic aspect, the transcriptomic analysis was conducted to construct a network of gene signatures that were regulated by the test samples. The genes with differential expression variance and gene pairs with differential expression covariance were identified by comparing the test prototype group with the UV reference group. In general, the network regulated by the USBT2627‐containing formula was identified to construct the perturbated network, which contained 887 genes. The network topology analysis showed that there were isolated subnetworks, and the biggest subnetwork contained 383 genes with 861 edges (Figure 6a). These genes in the differential network were significantly involved in pathways like endothelin‐1 (ET‐1) signalling pathway, melanocyte development and pigmentation signalling pathway, calcium signalling pathway, cytokine signalling pathway, FGF signalling pathway, etc. Figure 6b demonstrated the link between pathways and genes within the differential network, and half of the genes were involved in the above pathways. This comprehensive network illustrated the holistic effect of the USBT2627‐containing formula, not only regulating skin lightening–related pathways like the involvement of pigmentation‐related pathways but also oxidation‐related pathways, cytokine pathways and skin barrier–related pathways.

**FIGURE 6:**
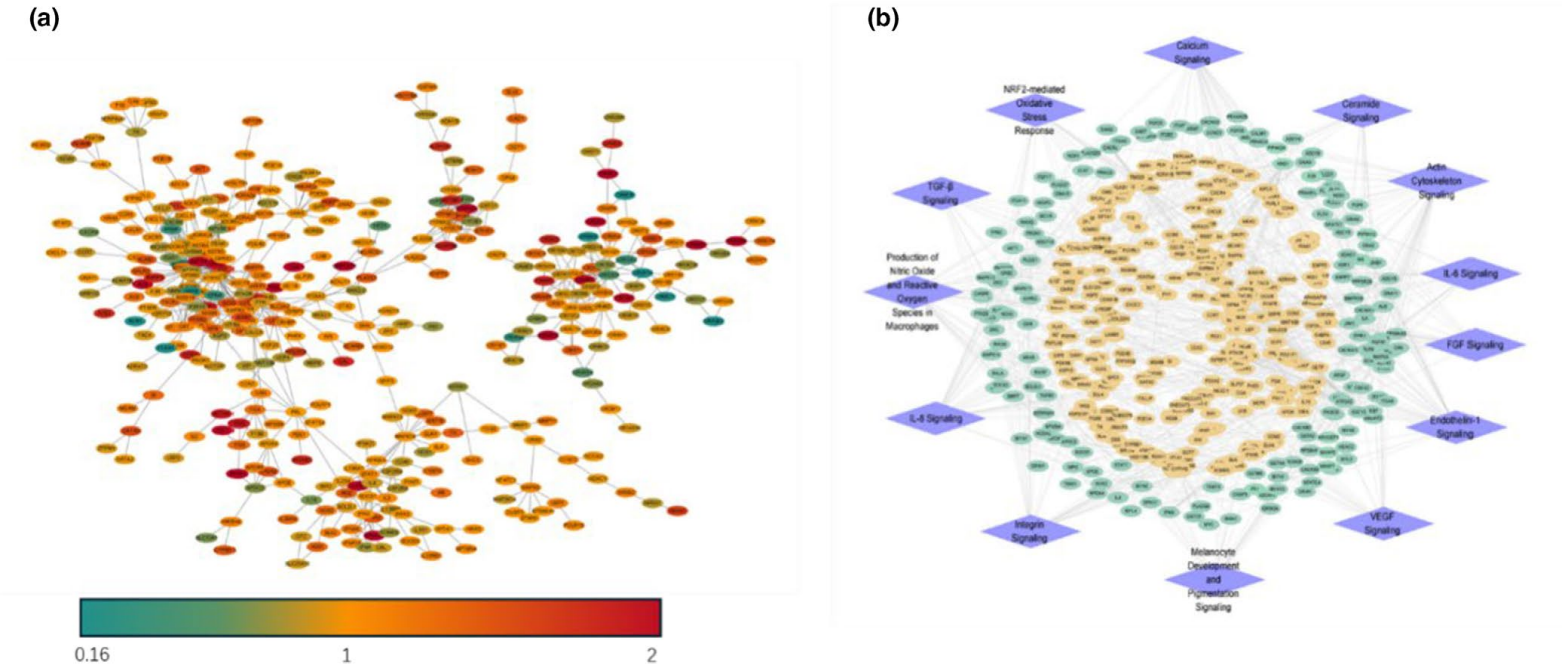
Differential network regulated by USBT2627‐containing formula. (a) The main subnetwork of differential network with gene indicated by fold change of differential expression analysis. (b) Allocated pathways on the differential network. Purple: pathways. Green: gene involved in pathways of interest. Yellow: other genes.

## DISCUSSION AND CONCLUSION

Hyperpigmentation is a skin disorder caused by overproducing melanin content in the skin, therefore, reducing melanin levels is critical for skin lightening. SBD, a classic skin lightening formula, has been used for over 500 years, its potential mechanisms underlying the skin lightening effect have been explored recently from in vitro or in silico approach. For instance, Li et al used docking and virtual screening to explore the link between TYR and the inhibitors in SBD [2]. However, no studies have yet utilized advanced skin models to explore the skin lightening efficacy of this formula with topical application, nor have clinical trials assessed the depigmentation effect of SBD incorporated in the cosmetic formulation. In this study, we provided a holistic evidence demonstrating (1) USBT2627 (the aqueous extract of SBD) decreased melanin content against UVB at a dose–response manner; (2) topical application of USBT2627 reduced the UVB‐induced melanin content of the pLSE model; (3) the USBT2627‐containing formula, a skin moisturizer formula containing 5% USBT2627 alleviated skin lightening relative parameters change (L*, ITA°) and redness induced by UV exposure compared with placebo; (4) the network pharmacology analysis implied the holistic effect of San‐Bai decoction; the indication was further validated by the differential network analysis on the transcriptome data between the 2 MED‐exposed skin samples with and without the USBT2627‐containing formula treatment.

SBD consists of four individual herbs with TCM compatibility rule behind, namely peony, poria, atractylodes and licorice. Phenolic compounds, recognized as antioxidants, are presented in each individual herb of SBD, particularly in licorice and peony [15, 16]. Melanogenesis pathway, where tyrosinase catalyses the hydroxylation of tyrosine and subsequently to dopaquinone further converting to melanin, is an oxidative process. Antioxidants have the potential to mitigate skin hyperpigmentation, thereby providing evidence for the lightening effect of SBD to a certain degree.

The in silico network pharmacology has been proposed to illustrate the interaction of containing components since it represents the relationship among ingredients and targets based on prior knowledge and includes the AI‐based relationships mining from omics data [6, 7, 17, 18, 19]. Here, the network pharmacology approach presented that SBD potentially regulated oxidation and inflammation processes. Our previous study carried out from the pLSE model proposed that there was a tipping point before critical transition state during pigmentation process, the gene changes exhibited a dynamic pattern where inflammation and oxidative pathway were involved at the early phase, and melanocyte development and pigmentation signalling pathway showed up late [10]. Therefore, we adopted the pLSE model to explore the potential efficacy of SBD in reducing UVB‐induced pigmentation.

Results showed that the water extract of SBD, USBT2627, prepared by the conventional water extraction method, significantly inhibited melanin content induced by UVB exposure in the in vitro model. Both systemic and topical applications were explored; the former study demonstrated a dose‐dependent reduction by USBT2627 (Figure 3a), while the latter study showed USBT2627 delivered a comparable efficacy to kojic acid, a well‐known skin lighting agent by acting directly on melanin synthesis [20, 21, 22], suggesting applying USBT2627 would be an efficacious approach.

Skin exposed to acute UV irradiation will induces various skin alterations in a dynamic and non‐linear fashion, including DNA damage, oxidation, and inflammation, apoptosis, cell death, melanogenesis [10]. Skin under UV challenge is well recognized as the standardized pigmentation in vivo model for the assessment of skin lightening efficacy of cosmetic products as mentioned in the Safety and Technical Standards for Cosmetics. Here, to explore the underlying mechanism for its preventive efficacy for skin lightening, we adopted the methods from public research [23, 24, 25]. In the clinical study, two dosages of MED were applied on Fitzpatrick skin phototype III individuals. It was clearly shown that 2 MED induced severe phenotypic changes, which is aligned with the widely accepted notion that higher UV dose, having a more pronounced impact on the skin, including increased skin blood flow, dilation of vascular structures and thickening of epidermis [26]. The phenotype changes over time under 1 MED and 2 MED were different as L* consistently decreased under 2 MED but rebounded at longer term after UV irradiation. This finding provides important significance for experiment design in future assessments.

In this study, the USBT2627‐containing formula showed significant improvements in L*, a* and ITA° at 10 days against 2 MED irradiation, compared with the placebo group, however, we did not find the same pattern after comparing the USBT2627‐containing formula and placebo group upon 1 MED irradiation, while it is worth mentioning that the USBT2627‐containing formula showed a significant alleviation in erythema score over the placebo group at the first day post 1 MED exposure. Relevant studies have shown that in human skin, compared with a single exposure to 1 MED, a single exposure to 2 MED results in a decrease in CD1a + LC (Langerhans cells) in the epidermis and an increase in CD11b + macrophage. The CD1a + LC is associated with the weakened ability of the skin to respond to antigens, while CD11b + macrophage is related to the induction of active immune suppression [27]. Another study involving 15 volunteers found that 2 MED irradiation induced significant inhibition of delayed‐type hypersensitivity [28]. Combined with our findings, we hypothesized that incorporating USBT2627 into a sunscreen formulation could provide an adjuvant role in treating post‐inflammatory hyperpigmentation and inflammaging.

Differential network analysis could present not only the change in individual genes but the gene–gene interaction, showing a panoramic overview of the biological systems, and has been widely used in disease conditions and biological systems response to external stimuli [29]. There were various approaches to identify networks, like co‐expression network [30, 31], or based on Gaussian Graphical Model [32]. Here, our differential network incorporated information of genes with differential expression, differential variance and differential co‐variance. This method captured not only the expression change but also the expression fluctuation and the co‐variance of gene pairs. Conventional analysis presented differentially expressed genes and their involved biological processes, which showed that keratinization and ET‐1 signalling pathway were involved by USBT2627‐containing formula‐regulated differential expression genes (Figures S7 and S8). The differential network analysis presented the gene–gene interaction change under the USBT2627‐containing formula treatment to demonstrate how this formula affected gene–gene interaction to interfere with biological processes. Our results showed that the USBT2627‐containing formula‐regulated multiple biological processes not only melanogenesis but also inflammation and oxidation to maintain skin homeostasis, echoed to the network pharmacology analysis, emphasizing its power in unravelling the complexity of TCM. Integration of network analysis with multi‐omics approach currently will be an effective and precise method to explore broad applications and for TCM modernization.

In conclusion, the present study demonstrated that the topical application of USBT2627 exerted potential skin lightening efficacy in reducing UVB‐induced melanin production in pLSE models. Statistically significant improvements were observed in skin lightening related features, including L*(*p* = 0.048), ITA° values (*p* = 0.022) under 2 MED irradiation and erythema (*p* = 0.016) under 1 MED, together with the mild change in a* (*p* = 0.052), suggesting its effectiveness of USBT2627 in mitigating UV‐induced skin changes. Network pharmacology implied that SBD may target multiple genes and proteins involved in oxidative response and immune regulation. Differential network analysis of transcriptomics data further suggested that USBT2627 may attenuate UV‐induced damage through multiple routes, including pathways related to keratinization, antioxidant action, and skin barrier function, underlining its multifaceted approach to skin health.

SBD, as a traditional medicine historically for skin benefit, possessing intrinsic complex requires a systemic approach to review and investigate its comprehensive mechanism. Here, network pharmacology, together with differential network analysis in the whole transcriptome gene expression profiling from clinical samples may offer valuable insights into the multifaceted actions of complex natural mix. Combining with the findings in 3D skin model and phenotypic change in clinical study, our results support SBD's potential application in modern dermatology. To further substantiate its skin‐lightening efficacy and validate mechanisms, additional experiments were required. Moreover, concepts emerging from the network analysis, such as the inflammaging or immune‐mediated pathways, will need a more complicated model, such as an immune‐competent skin model.

## AUTHOR CONTRIBUTIONS

**Rui Zhang:** Formal analysis, methodology, writing‐original draft. **Hong Zhang:** Formal analysis, investigation, methodology, software, visualization, writing – original draft, writing – review & editing. **Chengming Zhang:** Formal analysis, methodology, software, validation, visualization. **Wencai Jiang:** Data curation, investigation, methodology, project administration. **Luonan Chen:** Conceptualization, formal analysis, methodology, resources, software, supervision. **Yimei Tan:** Conceptualization, formal analysis, methodology, resources, software, supervision. **Ying Zou:** Investigation, methodology, project administration. **Xuelan Gu:** Conceptualization, data curation, funding acquisition, methodology, resources, supervision, writing – original draft.

## FUNDING INFORMATION

This research received no external funding.

## CONFLICT OF INTEREST STATEMENT

Hong Zhang and Xuelan Gu are currently employed by Unilever R&D Shanghai. This company had no role in the design of the study; in the collection, analyses or interpretation of data; in the writing of the manuscript; or in the decision to publish the results. Yimei Tan, Wencai Jiang and Rui Zhang from Shanghai Skin Disease Hospital received funding from Unilever R&D Shanghai for the execution of the clinical field work. Chengming Zhang and Luonan Chen received funding from Unilever R&D Shanghai for the network analysis. The authors declare no additional conflicts of interest.

## Supporting information


**Figure S1.** Experiment of design for 3D living skin equivalent models. In the systemic application experiment, pLSE models were exposed to 50 mJ/cm^2^ UVB irradiation once daily as the UVB group. In the active group, models were exposed to UVB irradiation once daily and USBT2627 was added into the medium starting from day 3 to day 6. In the topical application experiment, pLSE models were challenged with UVB once daily as UVB group. In the active group, the models were exposed to UVB irradiation once daily and USBT2627 was topically applied to the model surface starting from day 3 to day 6.
**Figure S2.** Schematic diagram of the implementation site in the clinical trial. In the clinical trial, the left and right buttocks of each participant were randomly designated for phenotype study and mechanism study.
**Figure S3.** Flowchart of the clinical trial study. This flowchart illustrates the sequence of events over the 16‐day clinical trial. UVR denotes ultraviolet radiation, MED denotes minimal erythema dose, AE denotes adverse event. Groups A to G represent different treatment conditions: A, No treatment; B, Placebo; C, USBT2627‐containing formula; D, Placebo; E, USBT2627‐containing formula; F, Placebo; G, USBT2627‐containing formula. Light blue indicates exposure to 1 MED, dark blue indicates exposure to 2 MED, and grey indicates that a biopsy has been performed.
**Figure S4.** Diagram for differential network methodology. Genes with differential expression (blue), differential expression variances (yellow) and differential expression covariance (purple) were identified. The differential network was composed of these identified genes.
**Figure S5.** UV absorption test of USBT2627, USBT2727‐containing formula. A: UV absorption profile of USBT2627‐containing formula and placebo in 1000x dilution. Blue line: USBT2727‐containing formula, Red line: placebo B: UV absorption profile of USBT2627‐containing formula and placebo in 400 ppm. Purple line: USBT2727‐containing formula, Red line: placebo C: UV absorption profile of USBT2627 raw materials. Blue line: USBT2627 in 1000 dilution (1000 ppm).
**Figure S6.** Changes of skin appearance under UV irradiation. A: a* value change from baseline (CFB). B: ITA° value change from baseline. C: L* value change from baseline. D: Erythema value change from baseline.
**Figure S7.** Top biological processes involved by differentially expressed genes of USBT2627‐containg formula against UV group.
**Figure S8.** Top pathways involved by differentially expressed genes of USBT2627‐containg formula against UV (IPA result).

## Data Availability

The data of this study is available from the corresponding author upon reasonable request.
