# Supplementary material for: Mechanisms underlying the protective effects of San‐Bai decoction against UV radiation on the skin
Source: Int J Cosmet Sci. 2026 Mar 23;48(5):1006–17. doi: 10.1111/ics.70093 (PMC13636175; doi:10.1111/ics.70093)
Supplement: Supplementary file 1 [file ICS-48-1006-s001.docx]

**Figure S1**

**Figure S2**

**Figure S3**

**Figure S4**

**Figure S5**

| **A**   | **B**   |
| --- | --- |
| **C**   |  |

**Figure S6**

**Figure S7**

**Figure S8**

**Supplementary methods**

**Light Source of UVR in clinical study**

For UVR minimal erythema dose (MED) determination above the waistline and erythema induction in the upper buttock region for each subject, the radiation source will be the SOLAR LIGHT model 601-300 (Solar Light Co., Philadelphia, Pennsylvania). This solar simulator generates a uniform collimated beam of 0.5 cm² (square for MED determination and a round area with an 8 mm diameter for UVR irritation) that closely matches the spectral characteristics of sunlight.

The artificial luminous source consists of a 300-watt xenon arc lamp, which emits a continuous spectrum from ultraviolet (240 nm) to infrared (> 800 nm). A highly regulated power supply ensures constant electrical power to the lamp, while a light intensity controller interfaces with the supply to maintain the long-term stability of the lamp's output, thereby ensuring consistent radiation levels for more precise exposures. The solar simulator is equipped with a dichroic mirror that transmits wavelengths from 290 to 400 nm, significantly reducing the visible to infrared output of the lamp. SCHOTT WG 320 UV blocking filters are employed to achieve a UVA + UVB spectrum and to filter out wavelengths below 290 nm. Infrared wavelengths are eliminated using a dichroic filter.

The luminous flux within the UVA + UVB spectral bands is measured in μW/cm² using a PMA 2100 SOLAR LIGHT radiometer, which incorporates photo-sensitive cells with optimal sensitivity to erythema-inducing wavelengths (UVA + UVB).

**Measurement of Minimal Erythema Dose**

The minimal erythema dose (MED) for an individual is defined as the lowest ultraviolet (UV) dose that results in the first visible, distinct erythema with well-defined borders, covering most of the UV-exposed area. The MED on unprotected skin, termed 'MEDu', is ascertained through the following method: a series of incremental timed UV light exposures are applied, with each subsequent exposure being 25% greater than the previous one. Erythema assessment at the exposed sites is conducted 16 to 24 hours post-irradiation.

To accurately determine the MEDu, it is essential to induce a degree of erythema that is noticeably more intense. The aim is to include exposures that result in no effect, while ensuring that the highest exposure that elicits a response is at most twice the energy of the minimal exposure that produces any effect.

The evaluation of the sites is performed to identify the minimum exposure time at a fixed energy level (expressed in seconds with units of J/m^2) that induces the first perceptible, unambiguous erythema with clear borders over most of the UV-exposed field, in accordance with the scoring system outlined below.

Table S1 Erythema in response to UVR irritation grading scales

| **Erythema Grade** | Skin reaction |
| --- | --- |
| **0** | no erythema |
| **1** | barely perceptible erythema |
| **2** | light red, marked erythema |
| **3** | dark red marked erythema |
| **4** | violet erythema |

Note: Half-point scoring may be used to distinguish between grades
